# Adaptation of a probabilistic method (InterVA) of verbal autopsy to improve the interpretation of cause of stillbirth and neonatal death in Malawi, Nepal, and Zimbabwe

**DOI:** 10.1186/1478-7954-9-48

**Published:** 2011-08-05

**Authors:** Stefania Vergnano, Edward Fottrell, David Osrin, Peter N Kazembe, Charles Mwansambo, Dharma S Manandhar, Stephan P Munjanja, Peter Byass, Sonia Lewycka, Anthony Costello

**Affiliations:** 1 Centre for International Health and Development, UCL, Institute of Child Health 30 Guilford St, London WC1N1EH, UK; 2 Umeå Centre for Global Health Research, Division of Epidemiology and Global Health, Department of Public Health and Clinical Medicine, Umeå University, SE 901-85 Sweden; 3 Baylor College of Medicine Children's Foundation Malawi; Private Bag 397, Lilongwe, Malawi; 4 Kamuzu Central Hospital Lilongwe, Department of Pediatrics, PO Box 149, Lilongwe, Malawi; 5 Mother and Infant Research Activities (MIRA), Kathmandu Medical College G.P.O. Box 921, Kathmandu, Nepal; 6 College of Health Science, University of Zimbabwe, Department of Obstetrics and Gynaecology, Po Box A178, Harare, Zimbabwe

## Abstract

**Background:**

Verbal autopsy (VA) is a widely used method for analyzing cause of death in absence of vital registration systems. We adapted the InterVA method to extrapolate causes of death for stillbirths and neonatal deaths from verbal autopsy questionnaires, using data from Malawi, Zimbabwe, and Nepal.

**Methods:**

We obtained 734 stillbirth and neonatal VAs from recent community studies in rural areas: 169 from Malawi, 385 from Nepal, and 180 from Zimbabwe. Initial refinement of the InterVA model was based on 100 physician-reviewed VAs from Malawi. InterVA indicators and matrix probabilities for cause of death were reviewed for clinical and epidemiological coherence by a pediatrician-researcher and an epidemiologist involved in the development of InterVA. The modified InterVA model was evaluated by comparing population-level cause-specific mortality fractions and individual agreement from two methods of interpretation (physician review and InterVA) for a further 69 VAs from Malawi, 385 from Nepal, and 180 from Zimbabwe.

**Results:**

Case-by-case agreement between InterVA and reviewing physician diagnoses for 69 cases from Malawi, 180 cases from Zimbabwe, and 385 cases from Nepal were 83% (kappa 0.76 (0.75 - 0.80)), 71% (kappa 0.41(0.32-0.51)), and 74% (kappa 0.63 (0.60-0.63)), respectively. The proportion of stillbirths identified as fresh or macerated by the different methods of VA interpretation was similar in all three settings. Comparing across countries, the modified InterVA method found that proportions of preterm births and deaths due to infection were higher in Zimbabwe (44%) than in Malawi (28%) or Nepal (20%).

**Conclusion:**

The modified InterVA method provides plausible results for stillbirths and newborn deaths, broadly comparable to physician review but with the advantage of internal consistency. The method allows standardized cross-country comparisons and eliminates the inconsistencies of physician review in such comparisons.

## Background

Cause-specific mortality data on childhood deaths are vital to identify health needs, compare patterns of death across populations, plan and monitor interventions, and inform policy [1-3]. In high-income countries, all births and deaths are enumerated through vital registration systems, and death certification is routine. In low-income settings, most births and deaths occur at home, death certificates are rarely available, and vital registrations are often inadequate or nonexistent [2-4].

Verbal autopsies (VAs) provide an alternative means of identifying probable causes of death through interviews with a close caregiver of the deceased, in which information about the circumstances, signs, and symptoms leading to death are gathered. VAs have limitations: they require recollection of events at the time of death, rely on understanding and reporting of signs and symptoms by interviewees, and may be influenced by interviewer skills. The data must also be interpreted to establish a diagnosis [5]. Conventionally, VA questionnaires are read by two or more physicians separately and one or more causes of death are attributed. A cause of death is established when physicians' opinions correspond; otherwise diagnosis is reconsidered and discussed with or without the input of an additional physician. If no agreement is reached, the cause of death is considered undetermined. Repeatability of this diagnostic process over time and in different settings is problematic, particularly when diagnostic criteria are not standardized amongst different clinicians [6-8]. In some situations, disagreement between physicians is such that a large proportion of causes of death remain indeterminate [7,9]. Moreover, the method is costly, time-consuming, and requires the involvement of physicians who are an already overstretched resource in low-income countries [6,10].

Despite these limitations, VAs are useful in estimating cause-specific mortality fractions (CSMFs) in population studies [6,8,11]. They have been used extensively in epidemiological studies, household surveys, and sentinel surveillance sites, and have been piloted in subsamples from sample registration systems. There remains a need to refine the technique to make it more comparable, repeatable, easy to apply, and cost-effective.

VA questionnaires devised by the WHO attempt to standardize the interview process, but more standardized approaches to interpreting VA data are needed. Hierarchical algorithms and computer programs based on logistic regression have been used, but they are difficult to standardize across cultures and age groups and can usually only identify single causes of death [12,13]. InterVA uses a probabilistic method and has been tested in a range of settings for deaths at all ages, across sexes, and for maternal deaths [14,15].

We describe the refinement and evaluation of InterVA to identify causes of death in the perinatal (stillbirths and neonatal deaths in the first seven days) and neonatal periods, using data from three different settings: Malawi, Zimbabwe, and Nepal.

## Methods

Based on Bayes' theorem [16], the InterVA model calculates the probability of a set of causes of death given the presence of circumstances, signs, and symptoms (collectively called 'indicators') reported in VA interviews. The method is described in detail elsewhere [10,17]. Briefly, a finite number of causes of death are assigned to a predefined matrix of estimated probabilities of occurrence. The presence of indicators (Table 1) modifies the predefined probabilities of each cause of death upward or downward using Bayes' theorem according to the formula

**Table 1:** InterVA indicators and cause of death categories.

Indicators		Cause of Death Categories
was this an elder 65+ years	any chronic/recurrent diarr (4+w)	Perinatal asphyxia
was this an adult 50-64 years	any abdominal swelling	Congenital malformation
was this a female 15-49 years	any vomiting	Prematurity
was this a male 15-49 years	any yellowness/jaundice	Tetanus
was this a child 5-14 years	any abnormality of urine	Pneumonia
was this a child 1-4 years	any urinary retention	Malaria
was this an infant 4 wks-1 yr	any haematuria	Measles
was this a neonate < 4 wks	any swelling of ankles/legs	Meningitis
was she pregnant at death	no bilateral swelling of ankle	Diarrhea
did pregnancy end within 6 weeks	any skin lesions/ulcers	Bloody diarrhea
did final illness last at least 3 weeks	any rash (non-measles)	Other acute infection
did final illness last < 3 weeks	any herpes zoster	Malnutrition
was death very sudden/unexpected	any measles rash	Kwashiorkor
was death during wet season	any excessive night sweats	HIV/AIDS related
was death during dry season	any excessive water intake	Pulmonary tuberculosis
was s/he in a transport accident	any excessive urination	Chronic infection
did s/he drown	any excessive food intake	Maternal causes
had s/he fallen recently	any acute fever	Acute respiratory disease (not pnem.)
any poisoning, bite, sting	any persistent fever (> 2 wk)	Chronic respiratory disease
was s/he a known smoker	any enlarged/swollen glands	Acute cardiac
any obvious recent injury	any facial swelling	Chronic cardiac
was s/he known to drink alcohol	was there a coma > 24 hrs	Stroke
any suggestion of homicide	any weight loss	Diabetes
any convulsions or fits	any anaemia/paleness	Malignancy
any diagnosis of epilepsy	any drowsiness	Liver disease
was the fontanelle raised	any delayed/regressed development	Kidney disease
was the fontanelle or eyeball sunken	any diagnosis of asthma	Disorders of the digestive system
any headache	any diagnosis of diabetes	Diseases of the nervous system
was there paralysis on both sides	any diagnosis of heart disease	Sickle cell anemia
any paralysis/weakness on 1 side	any diagnosis of HIV/AIDS	Transport-related accident
any stiff neck	any diagnosis of hypertension	Accidental poisoning
any oral candidiasis	been discharged from hospital very ill	Accidental drowning
any rigidity/lockjaw	any suggestion of suicide	Other accident
abnormal hair coloring	any surgery just before death	Homicide
any coughing with blood	any diagnosis of TB	Suicide
any chest pain	was s/he adequately vaccinated	
was there a cough for > 3 wks	any diagnosis of liver disease	**Additional indicators**
was there a cough for up to 3 wks	any diagnosis of cancer	**did baby have arched back after 2 days**
any productive cough	any diagnosis of stroke	**baby stopped sucking after day 3**
any rapid breathing	any diagnosis of measles	**did the baby die on day 1**
any breathlessness on exertion	any diagnosis of kidney disease	**did the mother fail to receive tetanus toxoid vaccine**
any breathlessness lying flat	any diagnosis of hemoglobinopathy	**did convulsions happen on day 1**
any chest indrawing	any diagnosis of malaria	**was there no cry/move/breath at birth**
any difficulty breathing	any delivery complications	**was baby's skin puffy/mushy at birth**
any breast lump or lesion	any heavy bleeding around delivery	**did the baby fail to cry at birth**
any wheezing	was there prolonged labor > 24 hrs	
any cyanosis	were there convulsions during delivery	
any abdominal mass	was the baby born early < 34 wks	**Additional causes of death**
any abdominal pain	was the baby small < 2500 g	**Fresh stillbirth**
any diarrhea with blood	was there difficulty breathing at birth	**Macerated stillbirth**
any vomiting with blood	any congenital malformations	
any acute diarrhea (< 2 wks)	was this a multiple birth	
any persistent diarrhea (2-4 wks)	any umbilical infection	

Indicators added following refinement of InterVA for neonatal deaths are highlighted in bold.

where p (C|I) indicates the probability of a cause of death (C) given the presence of the indicator (I) and p(I/!C) is the probability of I in the absence of C [10].

Probabilities of final-cause categories increase or decrease in relation to specific signs and symptoms reported in the VA interview. If symptoms are not reported, the probabilities do not change. The program is available online http://www.InterVA.net. Users can enter the data as single cases or in batches, and the model generates up to three causes of death and their respective likelihoods. Prior to the current study, the probability matrix consisted of 34 cause of death classifications and 104 indicators [10].

### Data sources

To explore the performance of InterVA in different settings, 734 stillbirth and neonatal VAs were obtained from rural areas of three low-income countries.

In Malawi (Mchinji District), 169 stillbirth and neonatal VAs were collected from 2004 to 2005, as part of a cluster-randomized study evaluating two community interventions to improve maternal and child health [18]. Although designed for the study, the VA questionnaire was comparable in structure and content with the subsequent WHO questionnaire [19]. Completed questionnaires were interpreted independently by two Malawian pediatricians, who assigned up to three causes of death on the basis of a hierarchical classification and algorithm [20]. They were able to use alternative diagnoses where necessary. Discrepancies were resolved by discussion and, if consensus could not be reached, the cause of death was recorded as indeterminate.

In Nepal (Makwanpur district), 385 VAs were collected from 2001 to 2003 as part of a cluster-randomized study of a community intervention to improve maternal and child health [21]. The questionnaire was again comparable with the subsequent WHO tool. Questionnaires were interpreted independently by two Nepalese pediatricians, who each assigned a single cause of death on the basis of the same algorithm used in Malawi. Discrepancies were resolved after review by a third physician.

The third data source included 180 neonatal deaths from Zimbabwe, identified as part of a maternal and perinatal mortality study conducted in 2007 and 2008[22]. Neonatal VAs were conducted using the WHO tool. Questionnaires were interpreted independently by two physicians, who each assigned a single cause of death using the International Classification of Diseases and Related Health Problems (ICD-10). Discrepancies were resolved after review by a third physician (Table 2).

**Table 2:** Characteristics of the three studies used as data sources.

	Malawi	Nepal	Zimbabwe
**Neonatal mortality rate**	27/1000[30].	33/1000[31]	24/1000[32]

**Study period**	1 year	3 years	2 years

**Number of VA questionnaires**	169	385	180

**Questionnaire**	Mixed open and closed questions	Mixed open and closed questions	Standard WHO tool incorporating open and closed questions (24)

**Interviewers**	5 lay Malawian interviewers with secondary education	Lay local field coordinators	45 midwife enumerators

**Physician review**	2 experienced local pediatricians Predefined algorithm 3 causes of death	3 experienced local pediatricians Predefined algorithm 1 cause of death	3 experienced local physicians International Classification of Diseases and Related Health Problems (ICD-10) 1 cause of death

### Refining InterVA

Initial refinement of the InterVA model was based on 100 (59%) physician-reviewed VAs from Malawi. The use of these data for refinement was pragmatic in that, at the time of refinement, they were the only data available. Data from the VA questionnaire were entered in the InterVA model, which assigned causes of death and associated likelihoods. The open histories, where the caregiver reported the events leading to death, were coded and also entered in the model. CSMFs obtained using the original InterVA and physician review were compared. CSMFs were calculated from the InterVA output as the sum of the likelihoods computed for each single cause of death category, divided by the sum of the likelihoods for all causes. For the calculation of CSMFs from physician-review data, if more than one cause of death was assigned, each was considered as a proportion of the total death. Therefore, if a single cause of death was assigned by all physicians, or if only one was available, it explained 100% of that death. If more than one cause of death was attributed, each contributed an equal proportion of the total 100%. For example, if both reviewing physicians assigned prematurity as a cause of death and one of them also assigned sepsis, then prematurity contributed 75% and sepsis 25% to the death. In this way, every available physician diagnosis contributed to the cause-specific mortality profile, avoiding a potential loss of information and bias that might have been introduced by using consensus diagnoses alone.

Fifty-four neonatal-death questionnaires were analyzed with the original InterVA model. Stillbirths were initially excluded, as InterVA was not designed to classify them. The results of this first analysis identified the need for greater differentiation in the model among causes of death in the neonatal period. The InterVA indicators and matrix probabilities were therefore reviewed for clinical and epidemiological coherence by a pediatrician-researcher (SV) and an epidemiologist involved in the development of InterVA (EF). Following this initial refinement, InterVA was evaluated by comparing case-by-case diagnoses with physician-assigned diagnoses for the same 100 VA cases, as well the population-level CSMFs. A process of refinement and comparisons with physician review was undertaken until InterVA elicited mortality profiles deemed by the researchers to be plausible and satisfactorily comparable to physician review.

### Evaluating the refined InterVA model

The modified InterVA model was evaluated by comparing population-level CSMFs derived from the two methods of interpretation (physician review and InterVA) for a further and hitherto-untouched 69 VAs from Malawi, 385 from Nepal, and 180 from Zimbabwe. A diversity of data sources was chosen to assess the performance of InterVA in a range of settings. Comparisons of population-level CSMFs were considered paramount as InterVA is intended as a public health tool for health monitoring and program evaluation, rather than for use in clinical settings. Nevertheless, individual level, case-by-case comparisons between physician diagnoses and InterVA were also conducted and the kappa statistic for interrater agreement was calculated to further evaluate the InterVA against the only available alternative method in our populations [23].

### Ethical considerations

The Maimwana study (Malawi) received ethical approval from the Malawi National Health Sciences Research Committee; the MIRA Makwanpur, Nepal, study was approved by the Nepal Health Research Council and the Institute of Child Health and Great Ormond Street Hospital ethics committees; and the Zimbabwe Maternal and Perinatal Mortality Study received ethical approval from the Medical Research Council of Zimbabwe (MRCZ/A/1368).

## Results

### Refining InterVA

InterVA was modified to include two extra cause of death categories: fresh stillbirth and macerated stillbirth. To define the stillbirth diagnoses and differentiate among possible causes of stillbirth and neonatal death, nine further indicators were added to the model. The resulting modifications to the specific indicators and cause of death categories included in InterVA are shown in Table 1. As these are extra entities in the model, they run in parallel to the existing indicators and causes without directly affecting them.

**Figure 1:**
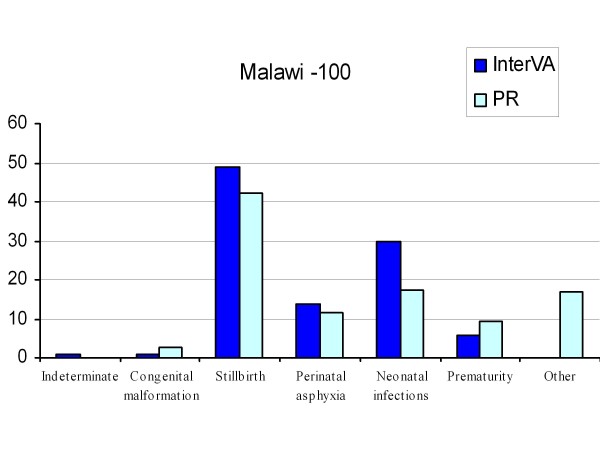
**Cause-specific mortality fractions from InterVA and physician review (PR) for the 100 VA cases from Malawi used to develop and refine the model**. Note to Figure 1: Other causes include "jaundice," "multiple pregnancies," "maternal causes," "hypothermia," and "hypoglycemia."

To compare the InterVA output and physician diagnoses in the three settings, some rationalization between the physician-assigned causes and the causes obtained from InterVA was necessary; therefore, causes of death not included in the InterVA classification were grouped as "other." Similarly, infectious causes of neonatal deaths, including sepsis, pneumonia, and meningitis were grouped together into an "infection" category, since the possibilities of clinically distinguishing them in newborn infants is difficult. There were no cases of neonatal tetanus. The resulting CSMFs for InterVA and physician review of the 100 VA cases from Malawi used to refine the model are shown in Figure 1. In 73% of cases, at least one of the InterVA diagnoses agreed with at least one of the physician diagnoses (kappa 0.60 (95% confidence interval [CI]: 0.57, 0.70)).

### Evaluation of the Refined InterVA Model

After refining the model, case-by-case agreement between InterVA and reviewing physician diagnoses, for 69 cases from Malawi, 180 cases from Zimbabwe, and 385 cases from Nepal, was 83% (kappa 0.76 (0.75 - 0.80)), 71% (kappa 0.41(0.32-0.51)), and 74% (kappa 0.63 (0.60-0.63)), respectively.

CSMFs derived from InterVA and physician review in Malawi, Zimbabwe, and Nepal are illustrated in Table 3. In Malawi and Zimbabwe, the rank order of causes of death was identical when derived from InterVA or physician review. In Nepal, the most common cause of death according to InterVA was perinatal asphyxia, while it was neonatal infections according to physicians. Prematurity was diagnosed more commonly by InterVA than by physicians in Nepal and Zimbabwe. InterVA detected a higher proportion of neonatal infections than physicians in Zimbabwe, but a lower proportion in Nepal.

**Table 3:** Comparison of cause-specific mortality fractions according to InterVA and physician review.

	Malawi 69 VA		Nepal 385 VA		Zimbabwe 180 VA	
	**Physician** **review**	**InterVA**	**Physician** ** review**	**InterVA**	**Physician** **review**	**InterVA**

**Stillbirth**	28.0	44.3	44.0	45.2	16.5	20.1

**Perinatal asphyxia**	18.8	19.4	21.5	26.4	11.3	9.9

**Neonatal infections**	23.3	26.0	28.0	20.4	30.6	44.5

**Prematurity**	10.4	7.5	3.1	6.5	18.2	23.9

**Congenital malformations**	2.2	1.3	0.8	0.9	1.3	0.4

**Other**	13.3		1.6		9.5	

**Indeterminate**	4.0	1.5	1.0	0.6	12.8	1.3

### Stillbirths

The proportion of total stillbirths identified by the two methods of VA interpretation was similar in all three settings. Data from Malawi and Nepal allowed for a more detailed comparison of the relative proportions of fresh and macerated stillbirths (Table 4).

**Table 4:** Fresh/macerated split of stillbirths from Malawi and Nepal based on interpretation by InterVA and physician review.

	Malawi 169 VA		Nepal 385 VA	
	**Physician** **review**	**InterVA**	**Physician** **review**	**InterVA**

Fresh	23.2	33.4	24.7	39.4

Macerated	4.8	10.9	19.2	5.9

Total Stillbirth	28.0	44.3	44.0	45.2

### Multicountry mortality comparison

**Figure 2:**
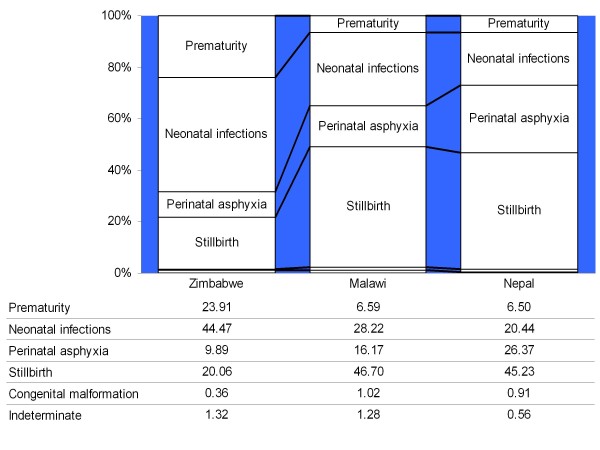
**Neonatal death cause compositions from InterVA interpretation of VA data from 169 deaths in Malawi, 180 deaths in Zimbabwe, and 385 deaths in Nepal**.

Considering the above evaluations and taking the refined model to be adequate for the purposes of characterizing cause compositions of neonatal mortality for population health planning and monitoring, a three-country comparison of neonatal cause-specific mortality was conducted (Figure 2). It showed some differences in cause compositions of neonatal deaths, particularly in Zimbabwe compared to the other two settings. In Zimbabwe, the proportions of preterm births and deaths due to infection were higher (44%) than in Malawi (28%) or Nepal (20%).

## Discussion

The deadline for the Millennium Development Goals (MDGs) is less than five years away and the need to quantify childhood mortality, understand its causes, and assess the effects of proposed interventions are central to MDG4. Neonatal deaths contribute about 40% of under-5 mortality globally [24]. A recent evaluation of the INDEPTH network of Health and Demographic Surveillance Sites [25] calls for all sites to use InterVA for coding of causes of death, since such approaches represent "the only viable strategy to produce timely and comparable cause of death statistics" [26]. Our study has revised the InterVA method for verbal autopsy to improve its ability to identify causes of stillbirth and newborn death and tested it in three populations.

In this study, physician review was used as a reference standard to compare InterVA. The use of physician review was the only alternative source of cause of death assessment for our study populations. This choice has limitations, however. Physicians are influenced by their experience, perception, and interpretation of local epidemiology [23,27]. Moreover, they mostly use the open history to reach a decision and may not account consistently for all the indicators. Sensitivity and specificity of physician review compared with hospital diagnosis in neonatal populations varied between 64% and 74% in a recent study [20] and concerns about inter- and intrarater reliability are well described [23].

An alternative to physician diagnoses is the use of hospital records. Hospital diagnoses have been used to establish sensitive, specific, and positive predictive values of VA diagnoses [8,12,20]. The main pitfall of hospital diagnoses in developing countries, particularly in rural settings, is that the CSMF of deaths occurring in hospitals are likely to be different from the ones in communities [23]. There is therefore the risk of increasing precision of an interpretative method, defined as its ability to reproduce hospital diagnoses in the population where it is tested. This would not necessarily produce results that are correct when used in populations where access to hospitals and health care is limited. Moreover, the ability to recognize, recall, and report signs of illnesses may be different among hospital users and nonhospital users.

The results of InterVA as compared with physician review showed an almost identical ranking of causes of death. However, differences exist. Some of these differences can be explained by the way the model was constructed. Prematurity, for example, was over-diagnosed by InterVA in Zimbabwe and Nepal. This probably resulted from using a dataset where clinicians were allowed more than a single cause of death to refine InterVA. In fact, when multiple causes of death are allowed, prematurity is more likely to be listed as a coexisting cause of death than when a single cause is selected [28]. The model did not include "other" as a cause of death and would have classified such causes of death in one of the available diagnoses.

InterVA over-diagnosed neonatal infections compared with physician review in Zimbabwe, while the opposite happened in Nepal. This inconsistency could be due to the interpretation of signs by different physicians. Alternatively, it could be due to the selection of *a priori *probabilities. Greater understanding of the way physicians decide to value or ignore signs and symptoms may help in future refinements and evaluations of InterVA.

Stillbirths were included for practical and public health reasons. Although globally there are about 3.2 million stillbirths per year, reliable statistics are lacking [29]. This information gap has to be addressed. About half of perinatal deaths are accounted for by stillbirths [29]. The refinements including stillbirths in the model eliminate the need to differentiate between live births and stillbirths before processing VA data, making the method more suitable for use in large surveys. The separation between fresh and macerated stillbirths is relevant, as prevention strategies are different. The comparisons between InterVA and physician review in Malawi and Nepal suggest that InterVA can differentiate the two categories, although, as with neonatal deaths, there may be room for further refinement.

Case-by-case agreement was moderate in all datasets, however it was lower for Zimbabwe compared to Nepal and Malawi. The new indicators and matrix probabilities have been chosen and modified on the basis of the personal experience of the researchers, and subsequently tested and modeled on a subset of the Malawi data. There is a risk, therefore, that the tool may be too closely modeled on a sub-Saharan African setting (although the results from Nepal do not support this) or on a particular research setup. In addition, the modifications have so far not been put to a panel of experts and may need to be subject to a wider consensus.

There may be important epidemiological and social explanations for the difference in the CSMF in Malawi, Zimbabwe, and Nepal. However, even if the interpretation of verbal autopsy data by InterVA was consistent, methodological variability in other aspects of VA may have contributed to the observed cause distribution. Indeed, the close comparability of CSMF between Malawi and Nepal may to some degree reflect common data capture processes that differ from those used in Zimbabwe. It is possible that in Nepal and Malawi, the populations were part of research areas and might have been sensitized to recognize, describe, and recall signs of neonatal diseases, while in Zimbabwe the community was part of a government surveillance and may have responded differently. Nevertheless, this is a reality of all VA studies conducted in research settings. Use of lay (in Malawi and Nepal) versus health-professional (in Zimbabwe) interviewers and their gender may also have had an impact on data capture. This highlights the need for further methodological research into the effects of other aspects of VA. It is likely that a number of strategies and international collaborations will be necessary to ensure the success of such investigations.

The modified version of InterVA for stillbirths and neonatal deaths produced plausible results when compared with physicians' opinions but had the advantage of being completely internally consistent, allowing standardized comparisons of data from different countries. Ultimately, standardized methods are essential and their application and evaluation in a wide range of settings is encouraged. Through wider application, the strengths and weakness of InterVA, and VA in general, will become more apparent, thereby better informing the application and public health utility of surrogate methods for measuring mortality in absence of vital registration systems.

## Competing interests

EF & PB contributed to this study with support from FAS, the Swedish Council for Working Life and Social Research (grant 2006-1512).

DO is supported by a Wellcome Trust Fellowship (081052/Z/06/Z).

## Authors' contributions

SV contributed to the setup of the Maimwana study in Malawi and to the formulation of the VA questionnaire used in Malawi and was involved in the adaptation and evaluation of the existing InterVA method, processing VA data from Malawi and Nepal, and drafting and reviewing the manuscript.

EF was involved in the initial development and testing of InterVA refinements and evaluation of the model for the current study, processing the Zimbabwe VA data, and drafting and reviewing the manuscript.

DO contributed to the setup of the Makwampur study in Nepal and to the formulation of the VA questionnaire in Nepal and Malawi. He provided the Nepal data and contributed to the interpretation of the results and revisions of the manuscript.

PNK was a PI of the Maimwana study and interpreted the VA questionnaires from Malawi.

CM was a PI of the Maimwana study and interpreted the VA questionnaires from Malawi.

DSM was a PI of the Makwampur study, interpreted the VA questionnaires from Malawi, and contributed to the final draft of this manuscript.

SPM was PI of the maternal and neonatal mortality survey in Zimbabwe and contributed to the interpretation of the study results.

PB devised the InterVA method to interpret VA and contributed to the interpretation of the results and revisions of the manuscript.

SL contributed to the setup of Maimwana study in Malawi, to the formulation of the VA questionnaires used in Malawi, and to the final draft of this manuscript.

AC ideated the Maimwana and Makwampur studies and contributed to the interpretation of the results and revisions of the manuscript.

All authors read and approved to the final draft of this manuscript.
